# Advancing Drug–Drug Interaction Prediction with Biomimetic Improvements: Leveraging the Latest Artificial Intelligence Techniques to Guide Researchers in the Field

**DOI:** 10.3390/biomimetics11010039

**Published:** 2026-01-05

**Authors:** Ridwan Boya Marqas, Zsuzsa Simó, Abdulazeez Mousa, Fatih Özyurt, Laszlo Barna Iantovics

**Affiliations:** 1 IT Department, College of Health and Medical Technology Shekhan, Duhok Polytechnic University, Duhok 42001, Iraq; ridwanmarqas@gmail.com; 2 Department of Software Engineering, Engineering Faculty, Firat University, Elazig 23119, Turkey; fatihozyurt@firat.edu.tr; 3 Department of Electrical Engineering and Information Technology, George Emil Palade University of Medicine, Pharmacy, Science, and Technology of Târgu Mureș, 540142 Târgu Mureș, Romania; zsuzsa.simo@umfst.ro; 4 Department of Computer Science, Nawroz University, Duhok 42001, Iraq; abdulazizmoosa93@gmail.com

**Keywords:** drug–drug interaction prediction, biomimetic machine learning, deep learning, graph neural networks, ensemble methods, knowledge graphs, pharmacokinetics, artificial intelligence, computational pharmacology, biomimetic computing

## Abstract

Drug–drug interactions (DDIs) can cause adverse reactions or reduce the efficiency of a drug. Using computers to predict DDIs is now critical in pharmacology, as this reduces risks, improves drug outcomes and lowers healthcare costs. Clinical trials are slow, expensive, and require a lot of effort. The use of artificial intelligence (AI), primarily in the form of machine learning (ML) and its subfield deep learning (DL), has made DDI prediction more accurate and efficient when handling large datasets from biological, chemical, and clinical domains. Many ML and DL approaches are bio-inspired, taking inspiration from natural systems, and are considered part of the broader class of biomimetic methods. This review provides a comprehensive overview of AI-based methods currently used for DDI prediction. These include classical ML algorithms, such as logistic regression (LR) and support vector machines (SVMs); advanced DL models, such as deep neural networks (DNNs) and long short-term memory networks (LSTMs); graph-based models, such as graph convolutional networks (GCNs) and graph attention networks (GATs); and ensemble techniques. The use of knowledge graphs and transformers to capture relations and meaningful data about drugs is also investigated. Additionally, emerging biomimetic approaches offer promising directions for the future in designing AI models that can emulate the complexity of pharmacological interactions. These upgrades include using genetic algorithms with LR and SVM, neuroevaluation (brain-inspired model optimization) to improve DNN and LSTM architectures, ant-colony-inspired path exploration with GCN and GAT, and immune-inspired attention mechanisms in transformer models. This manuscript reviews the typical types of data employed in DDI (pDDI) prediction studies and the evaluation methods employed, discussing the pros and cons of each. There are useful approaches outlined that reveal important points that require further research and suggest ways to improve the accuracy, usability, and understanding of DDI prediction models.

## 1. Introduction

The most significant pharmacological aspect related to DDIs is that they involve interactions, which often result in changes in safety, efficacy, or toxicity of therapeutics when deploying drugs together [1]. Most of these interactions have been associated with adverse drug reactions (ADRs) that could lead to an increased chance of hospitalization and a significant burden to the healthcare system [2,3]. DDIs have traditionally been detected via clinical trials, in vitro experiments, and post-marketing surveillance systems, including the Food and Drug Administration (FDA) Adverse Event Reporting System (FAERS) [4,5]. Nonetheless, this approach is slow, expensive, and small in both size and scope when applied to the increasing amount of pharmacological data and polypharmacy intricacy [2,3,6].

Recent developments in AI, namely, ML and DL, have been widely applied in pDDIs through the use of in silico approaches [7,8,9,10]. Initial ML models, such as LR, SVM, and random forests (RFs), perform reasonably well on small DDI datasets [4,7,11,12,13,14,15,16,17] but are limited in high-dimensional or graph-structured data settings [12,18,19].

Biomimetic computational methods are inspired by natural processes such as evolution, collective swarm behavior, or immune system adaptation. Examples include genetic algorithms for hyperparameter tuning or swarm-intelligence strategies for feature selection. Advanced architectures, such as graph neural networks (GNNs) or transformers, may only draw loose analogies to biological systems and are not inherently biomimetic unless specific components are directly modeled on biological systems. Biomimetic approaches enhance classical ML by remaining computationally efficient and effective in pDDI tasks [20].

DL architectures, including DNNs [9,17,21], convolutional neural networks (CNNs) [22], LSTM networks [23], and autoencoders [17,24], can detect latent patterns and dynamics of drug interaction data but need large volumes of training data and computing resources [25,26]. Neuroevolution strategies further enhance DL models by optimizing hyperparameters through mimicking natural adaptation mechanisms such as mutation [27].

Graph-based approaches, such as GCNs and GATs, offer clear advantages over simpler ML/DL methods for pDDI by modeling heterogeneous and large amounts of data of drugs, targets, and disease specifications as a connected set with network-like interactions, which are difficult to represent as traditional features [7,28,29,30,31,32,33]. Knowledge graph (KG) embeddings also consist of knowledge in the form of relational semantics between drugs and proteins [10,34,35]. Integration of biomedical text, chemical structure, and KGs using hybrid models improves model transparency and applicability [34,35,36]. These multi-entry relationships are effectively modeled with hypergraph neural networks, even in scenarios that involve novel drugs [37].

Transformer-based and hybrid approaches (DDI-Transform, Relational Transformers (RT), Fuzzy-DDI) [22,29,38,39,40,41,42,43,44] leverage biomimetic and attention-based strategies to generalize learned patterns. Immune-system-inspired attention mechanisms and pretrained tokenizers improve predicting heterogeneous pDDi data with adaptive biological memory processes [20,31].

Ensemble methods (EM) [11,16,18,21], which combine weak learners, are used to achieve greater robustness and generalizability in noisy data sets.

The integration of heterogeneous drug feature types at the network levels improves the accuracy of DDI prediction. Particle swarm optimization (PSO-FeatureFusion) [45], DL framework for Polypharmacy Side effects Prediction (DPSP) [46], and heterogeneous information network DDI (HIN-DDI) [47] each advance pDDI through multimodal feature integration, including distinct computational strategies. PSO-FeatureFusion fuses heterogeneous biological features using pairwise neural models. It is dynamically optimized via particle swarm optimization, providing accurate pDDI. DPSP creates similarity-based multimodal feature vectors about drugs that are aggregated into unified representations of drugs. These vectors are the input for the DNN that detects polypharmacy side effects across multiple datasets. HIN-DDI uses relational modeling by embedding drugs, proteins, pathways, and other biomedical data into a HIN to extract meta-path-based topological features that capture semantic interactions.

Physiologically based pharmacokinetics (PBPK) modeling is also a mechanism-based approach to modeling DDIs to describe absorption, distribution, metabolism, and excretion (ADME) processes in the human body. This approach simulates natural physiological ways to predict drug behavior [48,49,50]. These models have been effective in clinical and regulatory decision-making [51,52,53,54,55]. The use of PBPK models in the validation of DDIs [49] or simulation of DDIs [56] has been demonstrated in studies of oncology, infectious disease, and metabolic interactions [57,58,59].

With these developments, there are still difficulties to be concerned about. The quality of the data is not uniform, e.g, DrugBank [60], Chemical Database of Bioactive Molecules (ChEMBL) [61], Kyoto Encyclopedia of Genes and Genomes (KEGG) [62], Towards Understanding Side Effects of Drugs for Healthcare Data Analytics (TWOSIDES) [63], Side Effect Resource (SIDER) [64], and requires strict preprocessing, integration, and normalization [65,66]. The integration of the multi-omics data (genomics, proteomics, metabolomics) and biomedical text, which mirrors biological signal processing, is still limited due to complexity [67,68,69]. Moreover, most of the deep models cannot be easily interpreted in real clinical settings [24,30,70]. To fill this gap, explainable AI (XAI) methods are becoming popular, including feature attribution and graph-based attention mechanisms [28,30,71].

New directions in DDI research are indicated by recent developments in biomolecular crowding [69], positive-unlabeled (PU) learning [72], and diffusion models of drug design [73]. Multiview and substructural learning frameworks have also been revealed as promising, with studies such as Task-Specific Dual-View Substructural Learning Framework for pDDI (PEB-DDI) [74] and Dual-View Framework with Drug Association and Drug Structure for pDDI (DAS-DDI) [75]. Despite this, increased traction in terms of clinical relevance of algorithmic predictions is suggested by the recent addition of DDI-related clinical measures (Positive Predictive Value (PPV) and Positive Likelihood Ratio (LR+)) [36,48] in the evaluation of models.

In a path-breaking work described by Gao et al. [76], a multi-hop machine reading comprehension framework was presented that applied medical KGs to improve the explainability and reasoning capacity of DDI predictions. This study demonstrated how pathways in biomedical texts can be used to perform explainable inference. Experimental validation in pharmacokinetic testing provides solid claims of DDI potential of drugs, as illustrated by De Vries et al. [77] when they validated the DDI potential of Pritelivir using a clinical cocktail approach targeting CYP450 enzyme and drug transporters. Drug Sequence and Substructure features for pDDI (SSF-DDI), a DL framework introduced by Zhu et al. [78], manipulates both drug sequence information and molecular substructure features characteristics simultaneously, thereby demonstrating the combination of multiple types of molecular representations to be predictively efficient. Hanley et al. [79] used the PBPK modeling of brigatinib and noted the potential usefulness of such models in terms of estimating DDI risks in oncology.

The study presented in this paper is a synthesized review of AI-based technologies of pDDI that examines the theoretical advantages and disadvantages of ML, DL, graph-based, transformer, ensemble, fuzzy, hybrid, and PBPK approaches. This study suggests how to orient future research on the issue by suggesting what knowledge gaps might exist in terms of methods. It also proposes approaches capable of addressing them with an integrative, scalable, and explainable approach.

### 1.1. Objectives

This systematic review’s aims are as follows:

- Compare AI/ML approaches to DDI prediction over the family of methods: traditional ML and ensembles; DL (DNNs, CNNs, LSTMs, autoencoders); transformer-based methods; graph-based/GNNs; KG embeddings; hybrid/multimodal fusion; PU learning; fuzzy/rule-based systems; and PBPK models.
- Compare precision/recall/specificity (and optional PPV and LR+) across the datasets actually used in the nine included studies: DrugBank, ChEMBL, KEGG, FAERS, SIDER, TWOSIDES, Drug interactions and side effects (nSIDES), and FDA drug labels.
- Outline missed methodological opportunities and translational limitations (data standardization, class imbalance and leakage, interpretability/XAI, compute/scalability, incomplete external validation) and provide future research directions.

### 1.2. Contributions

This research adds value to the field through its following work:

- A full taxonomy of AI methods for pDDI (early ML/ensembles, new DL/transformers, GNN/KG, hybrid/multimodal, PU-learning, fuzzy/rule-based, PBPK) that are enhanced with biomimetic strategies and correlated with typical inputs, advantages, and disadvantages.
- Inclusion of transparently selected and excluded representative studies (2019–2025) guided by key PRISMA screening concepts to provide reproducible cross-method comparisons.
- Mapping to harmonized datasets and a preprocessing pipeline—including entity normalization, scaling, imputation, cross-resource integration, and pharmacovigilance signal filtering, with practice pointers.
- Uniformity of performance synthesis across large databases with common ML metrics, highlighting the limited—but important—use of PPV and LR+.
- Structured gaps analysis and research agenda (data quality/standardization; multi-omics and text combined analysis; XAI interpretability; scalable evaluation and leakage control).
- A strengths-and-limitations matrix by method family to inform model selection within the constraints of the real world (data modality/size, interpretability requirements, computational resources, intended clinical use).

### 1.3. Research Questions

To address the objectives, we refine the original research questions (RQs) to eliminate overlap and align with computational pharmacology best practices [5,6,7,8,9,12,13,16,17,18,19,22,23,24,25,26,28,29,30,34,35,36,38,39,40,41,43,44,48,49,50,54,58,59,60,61,62,63,64,65,66,70,71,72,79]:

- *RQ1 (Methods landscape)*: What are the most commonly used algorithm families in DDI prediction, including traditional ML, DL (DNN/CNN/LSTM/auto-encoder), transformers, graph models (GCN/GAT), KG embeddings, hybrid/multimodal, ensembles, PU-learning, fuzzy/rule-based, and PBPK, their theoretical foundations, and the data they require?
- *RQ2 (Performance and robustness)*: How do accuracy, precision, recall, F1, Receiver Operating Characteristics Curve–Area Under the Curve (ROC-AUC), Matthews Correlation Coefficient (MCC), and, where possible, PPV and LR+ change with the dataset (DrugBank, ChEMBL, KEGG, FAERS, SIDER, TWOSIDES, nSIDES, FDA labels) and with validation design (k-fold CV, temporal/stratified splits, external validation)?
- *RQ3 (Evaluation practice)*: What are the most common and the most suitable protocols (split strategies, imbalance handling, including PU-learning, leakage prevention, calibration) to achieve reproducible and clinically meaningful assessment?
- *RQ4 (Hybridization and interpretability)*: How do hybrid/multimodal models and KG-enhanced deep models compare to single-modality baseline models in accuracy and interpretability?
- *RQ5 (Translational readiness)*: What are current computational/methodological limitations (compute, data curation/standardization, explainability, external validity) that impede clinical translation, and in which directions (e.g., PBPK-aligned XAI, standardized datasets/benchmarks) is the field tradition most promising?

### 1.4. Rationale for Restructuring Research Questions

- Distinct identification of the problem statement, three objectives, and this study’s contributions are justified and conform to J Pharmacokinet Pharmacodyn’s guidelines for manuscripts [18] to enable the reader to understand the rationale for this study quickly.
- Elimination of redundancy in RQs (i.e., original RQ4 and RQ5 merged to RQ4) is relevant to the current and future Wiley Briefings in Bioinformatics reviews that contribute to model interpretability and knowledge integration [39].
- Implications: By formulating RQ5 around the issue of translational barriers, this review discusses a significant issue mentioned in ScienceDirect’s *Drug Discovery Today* [40] that links computational studies and clinical application.

## 2. Materials and Methods

Following key PRISMA 2020 guidelines [80] to ensure transparency and reproducibility, this systematic review comprehensively examines existing studies on pDDIs, analyzing the models and their implementations.

### 2.1. Coverage

In this review, the literature published between January 2019 and November 2025 (last search: 20 November 2025) is addressed. Web of Science, Scopus, PubMed/MEDLINE, and IEEE Xplore were searched, and forward/backward citation tracking of identified papers was performed. Only full-text, English-language, peer-reviewed articles were considered. Where there were multiple versions of the same work (e.g., a journal article), the most complete peer-reviewed version within the specified time frame was selected.

#### 2.1.1. Inclusion Criteria

The inclusion criteria were as follows:

- *Type and degree of study*: Proprietary research that proposes or tests a computational/AI model to predict or rank DDI or pairwise ADR risk (i.e., binary DDI presence, interaction type/mechanism, or severity/clinical impact at the drug-pair level).
- *Information that presents or exploits DDI evidence*: The study either
  - (a) applies known DDI/ADR data sources (e.g., DrugBank, TWOSIDES, SIDER, FAERS, ChEMBL, KEGG, FDA labels, Electronic Health Record (EHR)/claims)
  - or (b) offers a novel DDI dataset/resource based on curated evidence or pharmacovigilance/clinical evidence, which can be used to develop training/evaluation data.
- *Ground truth definition:* Ground truth can be labeled information, reported exchanges, or empirical measures of pair-wise alerts; these must be written precisely.
- *Results (what the outcome needs to be)*: The quantitative predictive performance on held-out/bit or cross-validation data is reported using at least one common measure (e.g., ROC-AUC, precision–recall–AUC (PR-AUC), F1, accuracy, precision, recall, MCC) on the pairwise task.
  - In multi-class tasks (e.g., interaction type or severity levels), per-class and/or macro/microscores are required.
  - For regression outputs (e.g., risk scores), we report $R^{2}$/MAE/RMSE or concordance.
  - Valuable metrics are often recorded clinically aligned with the metrics (e.g., PPV, LR+, calibration).
- *Evaluation design:* Details a validation approach (e.g., k-fold CV, stratified/temporal splits, or external test set) that adequately evaluates generalization and avoids label leakage.
- *Population and setting.* Small-molecule/biologic (human drug products). Only those studies that did not evaluate possible human drugs were excluded. Hence, only those studies were eliminated that involved assessing either non-human or non-therapeutic substances except when the results were subsequently applied to approved human drugs.
- *Time window/language.* The paper was published between the coverage window (Jan 2019–Nov 2025), in English, and is available in full text.

#### 2.1.2. Exclusion Criteria

1. Studies that are not directly related to pDDIs. These studies do not focus on pDDI and offer no additional methodological or conceptual insights.
2. Research that lacks empirical data or detailed methodological descriptions.

Our comprehensive study ensured the inclusion of relevant and significant research, providing a thorough understanding of the current methodologies and their practical applications in pDDI. Earlier work in 2019, ref. [17], presented novel neural network architectures using combined integrated similarity measures, which offer a promising route for pDDI. In 2020, ref. [9] focused on the scalability of ML models in such large-scale DDI datasets, while in 2022, ref. [8] employed EM methods that improved prediction accuracy across several datasets. These two studies were selected with rigorous application of ML models for DDI prediction through methodological transparency, diverse dataset usage, and equally robust validation techniques. In 2022 further improvements were reported: ref. [13] used FDA drug label data for their DL models, making real-world application possible, and the work of [24] presented their pioneering DL approaches.

**Figure 1:**
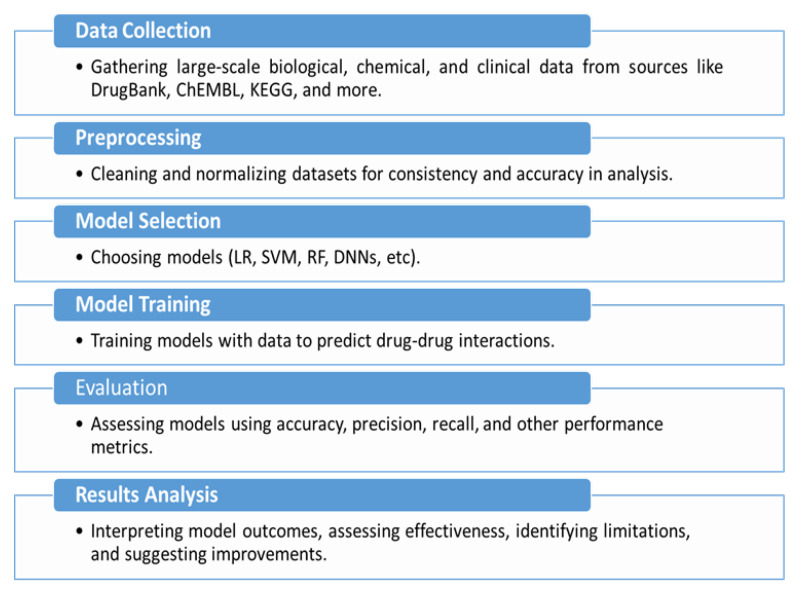
Schematic overview of the research methodology workflow.

Also, we considered the selection of techniques, which comprised methods, including graph-based methods and ensemble techniques, to look collectively at existing methodologies. Papers were also assessed in terms of the empirical evidence provided, methodological clarity, and contribution to advancing the field of pDDI and were determined to be important to include in this review. Figure 1 provides a schematic overview of the research workflow, illustrating the step-by-step approach of existing methodologies.

### 2.2. Critical Analysis

This subsection analyzes in more detail the advantages and disadvantages of different approaches, mentioning various studies that have demonstrated their possibilities and limits.

**Figure 2:**
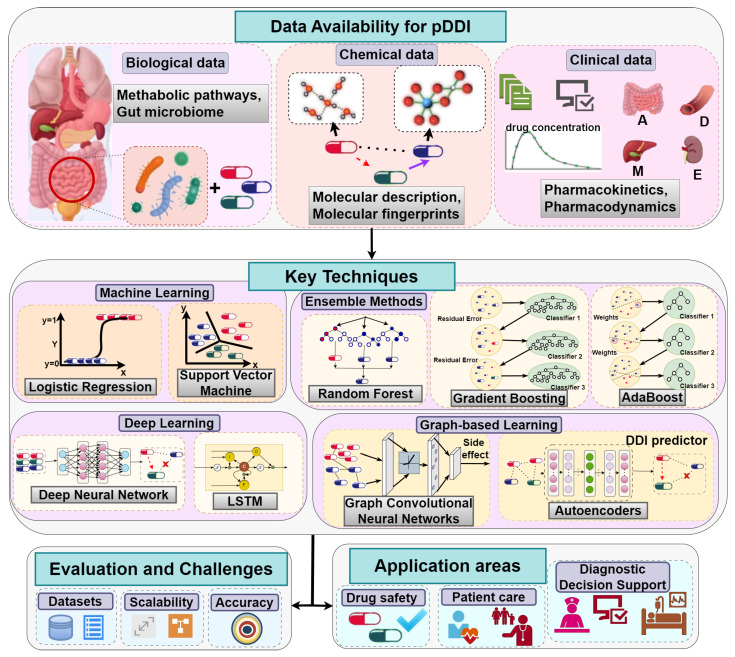
Overview of data sources and techniques for predicting potential drug–drug interactions.

Figure 2 provides a global view of the computational landscape for predicting potential DDIs. It emphasizes a wide range of data types used in this field, such as biological data (e.g., gut microbiome and metabolic pathways), chemical data (e.g., molecular fingerprints and structural descriptions), and clinical data (e.g., pharmacokinetics and pharmacodynamics). The figure also classifies the major computational approaches used, including classical ML algorithms (e.g., LR, SVMs), more recent approaches (e.g., DL, e.g., DNNs, LSTMs), EM (e.g., RF, gradient boosting), and graph-based learning models (e.g., GCNs and autoencoders). Moreover, it suggests the main assessment issues like reliability of data, scalability, and validity. It also provides examples of areas where the system may be used in practice, i.e., monitoring of drug safety, personalized patient care, and diagnostic decision support systems.

In Table 1, DL (particularly graph-based and KG-integrated approaches) generally yields high accuracy on the high-dimensional features that are complex, but it tends to need more data and computing in general. Classic ML models can be useful when speed is of value and when using small-data environments. Ensuring robustness and minimizing false positives via autoencoder-assisted and ensemble designs, as well as handling limited label availability, is supported through transfer learning (TL). Individual results using label text and chemical substructure features are not exhausted by combination, and there are no cases where they predict better than the combination does.

### 2.3. Identified Gaps in pDDI Research Studies

The most common difficulties in current research include the need for high-quality data and computationally extensive resources. In the following, different identified gaps are presented as a basis for future investigations. It is found through the systematic review that there are clear areas in pDDI research that need to be improved. Still, whilst ML and DL methods have advanced, the supply of quality data has not yet improved enough. Information from sources like DrugBank and ChEMBL is adequate but rarely complete and can vary a lot, inhibiting the accuracy and usefulness of models that make predictions [9,17]. In the coming years, researchers should rely on uniform data preprocessing to improve accuracy and consistency and maintain the completeness of their predictions. Biomimetic strategies could help guide the selection and weighting of heterogeneous data sources [7,27,42].

Currently, the effectiveness and size of current computational hardware remain an obstacle to successfully using graph-based neural networks and DL frameworks. Since these methods are relatively slow on current hardware, they are not typically used for immediate clinical assistance [21,28]. Finding better ways to apply these techniques, for instance, by using transformers—potentially through biomimetic-inspired optimizations such as neuroevolution or swarm-based architecture tuning for GNNs and transformers—will be a significant challenge in the near future [16,41].

It is also difficult for people to understand how models work. Though DL models such as DNNs and graph approaches give exceptional results, they are not easily understood by users and are therefore less useful in clinical applications, which is claimed in the findings by [8,24]. XAI techniques, such as attention and feature attribution, should assist significantly in gaining the trust and adoption of AI systems among clinicians and pharmacologists [29,41,67].

Connecting multi-omics data (including those from genomics, proteomics, and metabolomics) with chemical and clinical datasets is rarely undertaken because it is quite challenging and complex [19,68]. If the software used in drug studies can process numerous types of data simultaneously and efficiently—mimicking how biological systems integrate multiple signals naturally—it will significantly boost predictions. This can also broaden the range of models used in drug studies [69].

However, because of their high computational cost and implementation complexity, EM are used infrequently [18,70]. Experts should work on automatically finding the best hyperparameters and efficient ways to stack models—potentially leveraging biomimetic optimization approaches such as evolutionary search or swarm intelligence—so that using ensemble models remains efficient [73]. Fixing the highlighted gaps will be essential to improve the precision of the cardiology field. Developing more reliable, practical, and well-functioning pDDI prediction models will better ensure that patients are protected and receive better medical outcomes.

## 3. Analysis of the Research Methodologies Approached

An in-depth analysis is presented of how the studies were carried out, with an emphasis on their transparency, applicability, and possibility to be reproduced, and it incorporates ideas from recent advances in cheminformatics, NLP, and biomolecular modeling.

### 3.1. Transparency

We refer to pDDI as DrugDrug Interaction prediction, i.e., the computational prediction of whether, how, and with which clinical effect two drugs will interact upon co-administration. Transparency in this review is defined as a four-fold, DDI-specific construct encompassing the following: (i) model transparency, (ii) data/process transparency, (iii) experimental transparency, and (iv) clinical-mechanism transparency. This operationalization is a mix of previous pDDI efforts on interpretability/XAI, formal data curation and combination, transparent assessment, and mechanistic overlap (PBPK/KG) [24,36,48,65].

*i.*  *Interpretability/XAI (model transparency)*: Model transparency refers to how much can be predicted and how that can be traced back to inputs or learned correlations (e.g., attention maps, saliency/attribution, graph-attention weights) [24,28,30]; it copes with the notorious transparency in pDDI deep and graph models and is indispensable for clinical trust [24,70,71].
*ii.*  *Data and process transparency*: This dimension describes what information is used and in what forms (source identification, normalization, feature construction, scaling/imputation, KG building, signal filtering) [65,67,69]. Our common sources in the reviewed studies are DrugBank, ChEMBL, KEGG, FAERS, SIDER, TWOSIDES, nSIDES, and FDA labels [5,13,60,63,66]. Transparent reporting of these provides the ability to replicate labels/features and equitable comparisons between studies [42].
*iii.*  *Experimental openness (reproducibility)*: To ensure independent reproducibility of results, authors should report validation design (k-fold/stratified/temporal splits, external tests) and leakage control [25,36,66,72]. Standard ML metrics such as ROC-AUC, PR-AUC, F1, and MCC should be reported; if clinical metrics are provided, PPV, LR+ should also be provided, with calibration and uncertainty [9,36,48,49].
*iv.*  *Transparency of clinical mechanisms*: They must relate predictions to biologically or pharmacokinetically plausible mechanisms, such as through PBPK simulation/validation, which explains the changes in exposure [53,59]. Alternatively or in addition, drug–target–pathway chains that warrant pairwise risk can be revealed by knowledge-graph reasoning [26,28,30,34,35]. These mechanisms help adjust model outputs to inform safety and therapy optimization decision-making [48,50].

### 3.2. Suitability of Methods

A method is appropriate to pDDI when its inductive bias fits the data modalities (e.g., molecular graphs, label texts, time-series ADEs) and task definition (binary interaction DDI, interaction type prediction, interaction severity tier prediction, or continuous risk estimation) [8,10,25].

*A.*  A classical feature-based ML (LR, SVM, RF, gradient boosting.)
  LR, SVM, RF, and gradient boosting trees can comfortably handle tabular inputs, such as molecular fingerprints including Extended-Connectivity Fingerprint, diameter 4 (ECFP4), and Molecular ACcess System keys (MACCS); physicochemical descriptors (e.g., logP, molecular weight); bioactivity summaries; and label-derived indicators [12,16,17]. These models exhibit rapid training, easy calibration, and powerful baselines on small/medium datasets, but significant feature engineering is needed when higher-order nonlinearity is essential [8,12]. On small curated datasets, they tend to converge more quickly and calibrate better than deep nets, and deep nets take over as the feature dimensionality and volume of data increase [8,12]. Biomimetic-inspired optimization (e.g., genetic algorithms, swarm-based feature selection) is used to enhance hyperparameter tuning and feature selection efficiency [20,31,81].
*B.*  LSTM on temporal sequences of ADEs.
  A DDI classification network is based on an LSTM that accepts sparse and noisy ADE time-series as input, which are first compressed using an autoencoder [23]. This autoencoder-to-LSTM pipeline is suitable where temporal variations of exposure or concentration level bear a predictive value and the inputs are missing [23]. Compared to non-temporal, pure static baselines trained on snapshot features, the pipeline outperforms macro-F1. Temporal sequence modeling reflects how biological systems integrate signals over time [23].
*C.*  Deep stacked models (in ensembles, weighted voting).
  Outputs of DNN (structure features), CNN (substructure/sequence), and RNN/LSTM (temporal/sequence) models are combined through the meta-learner (stacking) or by weighted voting to capture jointly latent sources of errors [21]. Ensembles work well even with heterogeneous feature spaces and noisy labels, though at the expense of additional computational resources [21]. They perform significantly better than any single constituent deep model on average in AUC/F1 and minimize false positives, showing better generalization. A diversity-driven ensemble selection can mimic cooperative behavior in natural systems [21].
*D.*  GNNs and KG techniques.
  Drug structure: (i) Molecular graphs (atoms as nodes, bonds as typed edges) and (ii) DDI/biomedical KGs in which drugs, targets, pathways, and ADEs are related to each other using multi-relation edges. Topological signal represents relational information that a graph model uses, such as multi-hop paths, edge types, motifs/context, and neighbor-importance weights learned with attention [28,30]. Biomimetic inspiration, like immune-system mechanisms, can guide multi-hop message passing in adaptive network behavior [82]. GNs/GATs process molecular or DDI KGs at native resolution, with attention weights focusing on informative neighbors and relations [22,33]. The learned features are concatenated with KG embeddings (e.g., translational/rotational families) and passed into an MLP prediction/GNN to combine the relational semantic information with node/molecule features [26,29,30]. Graph models are favored when relationships themselves are predictive and when putative explanatory pathways are wanted [28,30]. The GAT tends to perform better at DDI relative to the vanilla GCN because it emphasizes informative neighbors and types of edges, and KG-integrated DL has lower false-positive rates relative to graph models that overlook the meanings of relationships [22,28,30,33].
*E.*  Transformer-based encoder (DDI-Transform; RTs; Pretrained Tokenizer and BiLSTM Model for pDDI (PTB-DDI)).
  Transformer encoders based on NLP that encode Simplified Molecular Input Line Entry Systems (SMILEs) or molecular substructures and relation sequences to predict DDIs [41]. Compared to typical sequence models, DDI-Transform has a superior predictive performance on DDI event prediction [38]. Relational/knowledge-aware transformers, e.g., encoding the edge types and KG signals, outperform the non-relational baselines [29,39]. Adaptive context weighting are biomimetic strategies that could enhance relational reasoning [81]. The TB-DDI model uses a pretrained tokenizer followed by a BiLSTM that serves as a computationally economical sequence baseline, since full transformers are computationally expensive [43].
*F.*  TL and PU learning.
  We evaluate K-dimensional representations in the context of TL, which pretrains on a large source dataset and fine-tunes on a small target dataset, which improves performance under label scarcity compared to training from scratch [70]. PU learning makes explicit use of unlabeled pairs to help combat the sentence-favored negatives, and it generalizes better at a large scale than negative sampling naively [72].
*G.*  Mechanistic PBPK.
  PBPK attains a mechanistic basis to explain and predict DDIs at the enzyme/transporter level and exposure changes. It simulates physiological ADME processes with AI predictions to provide mechanistic interpretability [48,50]. PBPK introduces clinical-mechanism transparency that can be applied in the explanation of the high-risk pairs or confirmation of AI predictions within contexts of dose and labeling [7,53,59,79]. PBPK is complementary to ML/DL screening as it has a physiological basis, and ML/DL has scalable discovery [48,49,50].
*H.*  Multimodal Integration of graphs, text, and molecular data.
  The King–Young-based multimodal DDI prediction framework’s representative pipelines jointly encode label/literature text (NLP/transformers), molecular graphs/ fingerprints, and KG relations; embeddings are concatenated or cross-attended and scored with an MLP/GNN [34,35,36]. The multimodal combination performs better than the single-modality baselines that were trained using a single modality (text or structure) and, unlike these baselines, also increases interpretability [34,35,36].

ML based on features is suitable for small to medium tabular environments and serves as a calibrated reference [8,12]. Graph and transformer families are better suited when relational or long-range molecular context is a risk driver, and attention/path rationales should be reported [22,29,33,38,41]. LSTM models are suitable for capturing temporal ADE signals, even in the presence of missing data [23]. Ensembles regularize performances on the heterogeneous inputs [21]. TL and PU are suitable when labels are scarce or biased [70,72]. PBPK is suitable when applying mechanistic explanation and validating high-stakes predictions [48,49,50,53,54,55,56,57,58,59,79].

### 3.3. Reproducibility

pDDI-specific definition: In pDDI, reproducibility would imply that given a defined snapshot of the input data, the same labels, preprocessing, split files and seeds, hyperparameters, code, and environment, a different group of researchers should be able to rerun the stated pipeline and generate the same numerical results [25,36]. Replicability among analogous data implies that the pipeline reproduces statistically analogous topics on a new snapshot of a similar source family following the stated preprocessing [36,42]. External validity (transportability) indicates performance is consistent across dissimilar datasets (e.g., train on DrugBank, test on TWOSIDES or nSIDES) with respect to a prespecified evaluation procedure [36,48,49,63,66]. Strict reproducibility on the same dataset version with public publication of code, configuration files, frozen split indices, random seeds, pinned packages/environment, preprocessing scripts, and any pretrained tokenizers/embeddings/weights are required [25,36,42,65,66,67,69]. Replicability is then determined by rerunning against a subsequent release of that resource (e.g., against a later DrugBank version) and checking overlap of confidence intervals or a preset non-inferiority level [36,42].

*A.*  Basic reporting criteria pDDI.
  - Provenance and versioning of data. Identify each source and its version/release/ date (DrugBank, ChEMBL, KEGG, FAERS, SIDER, TWOSIDES, nSIDES, FDA labels) and note the time of downloading/exporting and the terms of use [5,13,60,61,62,63,64,65,66,67].
  - *Negative labeling*. Discuss deriving positives and generation of negative/non-labeled pairs; where no true negatives are available, use PU or controlled sampling learning [36,72].
  - *Preprocessing pipeline*. Demonstrate how to entity normalize/map, build features, scale/impute, de-duplicate, build a KG, and signal grade on pharmacovigilance data; release scripts where available [65,66,67,69].
  - *Split design and leakage control*. Report whether the split is at pair level, at drug level, stratified, or temporal; explain the choice and publish split files to avoid label leakage [25,36,66,72].
  - *Metrics and uncertainty*. ROC-AUC, PR-AUC, F1/MCC, and when available PPV/LR+, as well as calibration and confidence intervals (e.g., bootstrap) [9,36,48,49].
  - *Code and environment*. Train/inference code (including random seeds), locked environment (needs/conda), and any non-deterministic operations [42].
  - *Model artifacts*. Include trained weights, tokenizer/vocabulary, and needful KG embeddings to make sure that runs are reproducible [42].
*B.*  Recommended checks.
  - *Minor rerun*: Recompute all measures on the frozen splits, seeds, and environment; make sure that everything matches in approximation up to numerical tolerance [25,36].
  - *Split-swap*: Compare drug-level versus random-pair splits to prevent information leakage from inflating results [36,66].
  - *Temporal holdout*: When asserting prospective benefit, testing after year t after training will be based on prior training data [36,48,49].
  - *Transversal validation*: Train on one resource (e.g., DrugBank) and test on another (e.g., TWOSIDES/nSIDES) to establish transportability [36,63,66].
  - *Noise/PU sensitivity*: Exploit label noise and PU settings to represent negative or uncertainty in negatives [72].

### 3.4. Datasets and Preprocessing

The existence of reliable and relevant data is critical for successful modeling of DDIs, as this data supports our understanding of drug effects, potential side effects, and how drugs work together at the chemical level. Datasets applied in DDI research have different structures, sources, and content. Breaking down the structures by pharmacological, ADE, and biochemical types can help to understand how suitable they are for modeling certain problems. In Table 2, the different datasets are matched to their types, content, and intended uses.

#### Link to DDI Modeling

Modeling DDI is improved when various datasets are integrated into ML and DL models. Some examples follow:

- Predicting the mechanism of action can be performed with the information available in ChEMBL and KEGG at pathway and target levels.
- By using FAERS and TWOSIDES, accurate validation and successful detection of any noise are possible for adverse DDI cases.
- DrugBank enables modelers to integrate chemicals, pharmacokinetics, and interaction data into neural networks by using multiple data types.

Both refs. [7,10] have shown that putting structural and clinical data together helps provide a more realistic view of drug interactions. Additionally, as part of graph-based reasoning, ref. [29] used SIDER and FAERS to assess the accuracy of predicted side effects. Consequently, the first stage in DDI work should always focus on understanding each set of data before filtering, so its specific pieces of information are structured for mechanism, target interaction, adverse signal, or pathway.

### 3.5. Preprocessing Steps

PDDI’s importance in medicine requires preprocessing that is tailored to pharmacology and highly precise. DrugBank, ChEMBL, FAERS, TWOSIDES, and KEGG are biomedical databases, and their raw data are often inconsistent, noisy, and vary significantly. Preprocessing steps for DDI, designed with the help of reviewed studies and references, are shown in the diagram below.

1. Data cleaning (entity normalization).
  - Frequently, drug names use different spellings, by brands, come in different salt forms, or are listed as synonyms in medical databases (like ‘acetaminophen’ and ‘paracetamol’ or ‘ibuprofen sodium’ and ‘ibuprofen’). Such entities have to be linked to the same standard, for example, Internal Nonproprietary Names (INN) or DrugBank IDs, by using controlled vocabularies.
  - Medical Dictionary for Regulatory Activities (MedDRA) coding hierarchies were used to resolve when side effect terms differed (like using ‘nausea’ instead of ‘feeling sick’).
  - If an entry had been given in multiple units or if there was a duplicate, these were either adjusted or removed following the approach taken in FAERS and SIDER curation guides [42,65].
2. Feature normalization and scaling.
  - Molecular weight, logP, and half-maximal inhibitory concentration (IC50) results from DrugBank and ChEMBL were scaled back so they do not go beyond the data range. This prevents large pharmacokinetic influences from stopping the model from being properly trained [5,67].
  - Researchers reduced the redundancy and improved learning in high-dimensional chemical data (such as RDKit fingerprints) using Principal Component Analysis (PCA) or t-distributed Stochastic Neighbor Embedding (t-SNE) methods [41].
3. Missing values (imputation strategies).
  - For missing bioactivity data in ChEMBL or incomplete pharmacokinetic profiles in DrugBank, mean or k-nearest-neighbor (KNN) imputation methods were used depending on the data sparsity.
  - For categorical labels (e.g., ADE presence in FAERS), mode-based imputation or flagging as ‘unknown’ was preferred over deletion to avoid information loss in rare drug–event associations [42,66].
4. Data integration.
  - Information gathered from DrugBank and KEGG needed to be aligned with drugs by using identifiers common to both datasets. To relate drugs to targets, pathways, and outcomes, authors made use of tools such as International Chemical Identifier (InChI) keys, SMILES strings, or Universal Protein Resource (UniProt) IDs [62,69].
  - Data schema transformations were required to standardize formats across sources—tabular activity matrices from ChEMBL, JSON-based pathway maps from KEGG, and XML entries in DrugBank were all reformatted to match the ML pipelines [42,73].
5. Adverse event signal filtering.
  - In post-market surveillance datasets like FAERS and TWOSIDES, false-positive ADE signals caused by reporting biases were mitigated using disproportionality analysis (e.g., proportional reporting ratios or empirical Bayes scores) [42,66].

**Figure 3:**
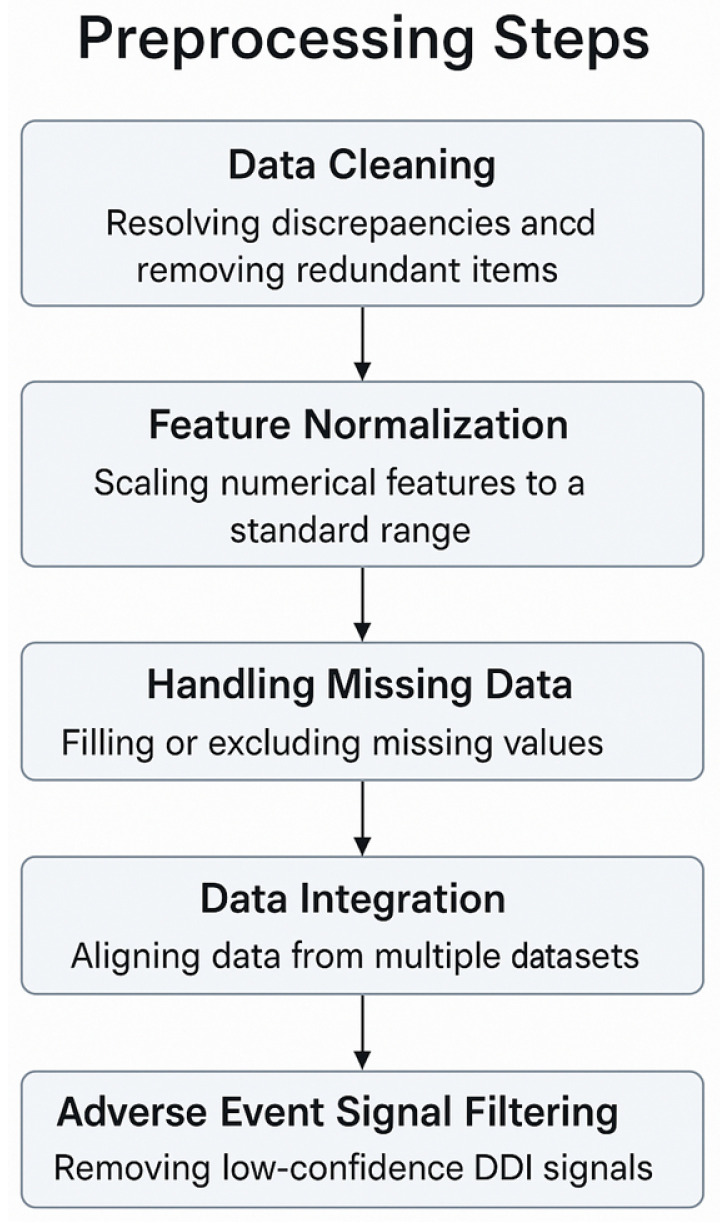
Preprocessing steps for pDDI modeling.

The way you preprocess data for a specific domain can have an increased impact on the accuracy, clarity, and capability of the model to work in different situations. If pharmacological inconsistencies are addressed, model factors are divided for fairness, and missing or unclear data are handled correctly, researchers’ results become more genuine. When data is not optimized, models are at a higher risk of error, mistakenly fit to the data, or lack usefulness for real-world pharmacovigilance tasks. These considerations and the main preprocessing stages used in pDDI modeling are illustrated in Figure 3.

## 4. Results and Discussion

### 4.1. Overview of Models Applied for pDDI and Related Biomimetic Considerations

The integration of ML, DL, and PBPK modeling has led to fast progress in pDDI. All of these approaches are different in how they work, the data they need, and how good they are at making predictions. ML and DL models have become popular.

Often, XGBoost and ensemble learning are chosen in pDDI since they are efficient and easy to understand [11,16]. Still, they have difficulty modeling interactions between drugs that are not simple and straightforward. Several DL models, for example, GCNs, transformers, and hybrid architectures, have improved the accuracy and scalability of drug discovery by encoding both molecular information and drug interaction networks. For instance, ref. [38] introduced DDI-Transform, a transformer model designed for pDDI, which performed better in prediction tasks [38]. According to [25], comprehensive DL methods are better at handling various drug data for pDDI. Ref. [34] introduced the Subgraph Enhance model for pDDI (SubGE-DDI), which combines biomedical data mining with KG to make predictions on pDDIs more precise. Biomimetic principles, such as neuroevolution or adaptive attention, could also optimize these models by mimicking natural learning [27].

GNNs and KG methods model drugs and their relationships as graphs, so they can examine the connections among them. Ref. [76] used multi-hop machine reading comprehension to help explain the predictions for possible DDI in medical KGs. The Graph Attention-based Deep Neural Network (GADNN) was developed by ref. [28] to connect various drug-related features by using a GAT. The team of [30] applied explainable GCNs to increase the transparency of pDDI models in medicine. According to [35], Domain-Invariant Substructure Interaction Learning for pDDI (DSIL-DDI) is a framework that helps predict pDDI of various drug pairs by dealing with common substructure patterns. These approaches reflect the relational organization observed in biological systems [35].

The use of pretrained components has boosted the study of pDDI methods. The authors in [43] used pretrained tokenizers together with BiLSTM networks and named their model PTB-DDI for making effective predictions about sequential interactions. According to [22], the Hierarchical Triple-view Contrastive Learning framework for pDDI (HTCL-DDI) is a hierarchical contrastive method that helps capture many drug aspects and achieves better results in pDDI prediction. Ref. [44] used fuzzy logic in Fuzzy-DDI so that the system could handle uncertainty in queries, which made the predictions more resilient in pDDI.

By means of PBPK modeling, the physiology of a drug is simulated to help understand how it is absorbed, distributed, metabolized, and removed from the body. Last year, Perrier et al. relied on PBPK models to estimate the effect of ziritaxestat on both enzymes and transporters [48]. In [49], the study explored the pharmacokinetics of imatinib and its metabolite to correctly judge the risk of drug interaction in oncology. In a number of studies [6,50,56], researchers used PBPK for modeling possible drug interactions of pH-sensitive drugs, antibiotics, and drugs used for cystic fibrosis. Further investigation of how enzymes cause pDDI is conducted in the study by [51], which revolves around CYP450 isoforms.

Adding clinical information and pharmacogenomics to the process boosts how accurate pDDI predictions are. In ref. [52], the researchers analyzed how genotypes in the CYP3A family affect the interaction between steroids and tacrolimus, proving that variations in genotypes can affect pDDI. By working with cocktails, ref. [77] examined effects of transporters and CYP450 enzymes on drugs, using top-reported drug interactions to support clinical outcomes.

For pDDI models to be reliable, researchers use important resources such as DrugBank, ChEMBL, KEGG, FAERS, SIDER, and nSIDES. Using standard figures and indicators makes it easier to compare different companies, according to [8,9]. Improvements in data construction and pretraining, as suggested by [36,75], improve the models that are better at working on different tasks [32].

Difficulties encountered in pDDI prediction occur in relation to the understandability of the prediction models, their handling of many kinds of data, and their use in clinical practice. Approaches that improve how easy a model is to understand [30], methods that integrate various data streams [34], and ways of learning from any domain [35] are all promising. Connecting what is possible in research and what can be used by doctors is still a main goal for everyone [48,50]. Diffusion models are a new approach mentioned by [73] that might lead to predicting pDDI in drug development processes.

In [42], ways to use NLP for protein–ligand analysis are reviewed, a key aspect of learning how pDDI works in molecules. They point out that language models may identify significant biochemical connections from texts to support predicting how people will respond. Also, ref. [41] looked at transformer models in cheminformatics, showing how they can better encode the structure and properties of drugs for improved prediction outcomes. Ref. [38] presented DDI-Transform, a transformer model that focuses on DDI events and achieved the best outcomes in studying how drugs affect each other’s reactions. In their study, ref. [26] examined DL applications used in pDDI and pointed out that transformer and graph-based models are the most popular for recent research. Last year, ref. [78] shared their SSF-DDI model that integrates both the drug sequence and substructure using DL to stress the significance of using both types of information for correct pDDI predictions. Work by [33] presented GCN-BMP, which uses a GCN to learn about molecular interactions, proving that such approaches are useful for DDI prediction. In 2024, a team of researchers presented PEB-DDI, a framework that uses dual-view substructures to improve the results in the related task [74].

Kim and Nam presented DeSIDE-DDI (2022), a model focused on DDIs and linking these interactions to the genes that respond to drug exposure [71]. In [72], the researchers came up with DDI-PULearn, a PU approach that allows predicting pDDIs in large-scale studies by dealing with the problem of incomplete negative labels, which could be addressed with adaptive pattern recognition or evolutionary tuning.

PBPK modeling keeps offering valuable explanations and clinical evaluation for predicting pDDI. Refs. [53,54,57,79] described the use of PBPK modeling to assess the interaction of sparsentan, brigatinib, maribavir, and adefovir with other drugs. They show how enzymes, transporters, and different physiological settings can impact the movement of drugs, adding extra details to what data alone can explain.

Purohit and colleagues (2025) explored how ritlecitinib works with the cytochrome P450 enzymes and their participation in drug–drug interactions [55]. Through a simulation, ref. [58] pointed out how physiological changes related to pregnancy affect efavirenz-dolutegravir interactions. In [59], the researchers studied the effects that CYP3A inhibitors have on venglustat pharmacokinetics, which they studied using both computer simulation and experiments. To spot DDIs in spontaneous event reports, ref. [3] made use of signal detection tools and checked whether the events were biologically possible. As a result of this process, data from actual cases are applied to check pharmacovigilance, and the model’s predictions are confirmed.

Also, research using hybrid and new approaches is important. The authors of the study in [69] examined developments in biomolecular crowding and condensation and their influence on DDIs when it comes to intracellular drug behavior. In addition, refs. [41,42] pointed out that combining chemical, biological, and textual data using transformers can make the predictions more accurate. It is explained by [53,57,79] that both ML/DL and PBPK models are helpful when used together for drug interaction assessments.

**Figure 4:**
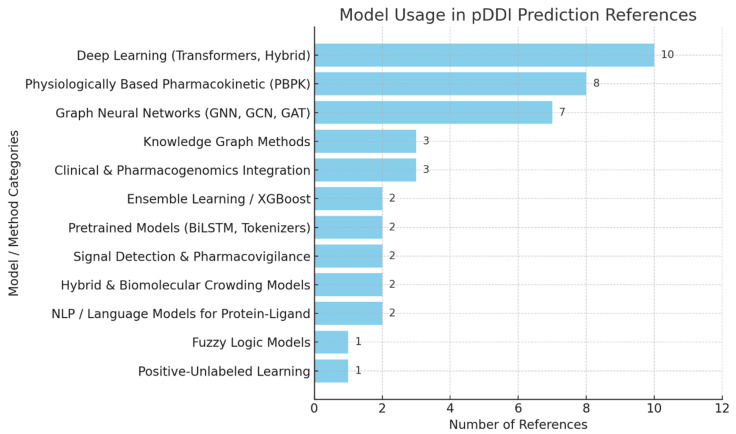
Model usage in pDDI references.

Figure 4 shows which computation models and methods are most frequently used in recent pDDI studies. Transformers and hybrid models (10) are mentioned most in the literature, indicating that they are becoming more important for correctly representing the interactions among drugs.

A few studies confirm that PBPK models play a key role (eight references) in simulating drugs in the human body for better interaction estimates. Earlier studies based on GNNs and graph methods are included (seven references). Some other approaches, such as using KGs, combining clinical and pharmacogenomic elements, and working with AI ensembles, are moderately used since they boost prediction performance. Pretrained networks, signal detection, fuzzy logic, and PU learning are not common solutions, but they offer useful features in dealing with different pDDI situations, many of which emulate natural processes such as adaptation and relational learning [51,52].

Overall, it seems that combining sophisticated DL neural architectures with pharmacokinetic models, as well as graph and integrative data methods, leads to better results and improved clinical use of pDDI prediction. In Table 3, the analysis of several pharmacological models for detecting DDIs shows how changes in ML and added data have led them to become more powerful and effective. RFs and similar methods, such as XGBoost and CatBoost, give dependable and scalable baseline performance, reaching an accuracy of about 85–90% for data on molecules and biology.

Meanwhile, GNNs and transformer models give better results, achieving an ROC-AUC higher than 0.90. These models are excellent to manage pharmacological information involving molecules, the genome, and medical publications. Their capacity to gather different kinds of data from DrugBank, SIDER, KEGG, and TWOSIDES improves their performance and helps them predict better outcomes. Mixing KGs, biomedical text, and different types of data has become the main approach in DDI prediction, making it more accurate and clearer. Still, PBPK models, and not only AI but also simulation of drug metabolism and interactions, are vital for the success of clinical translation.

In conclusion, these advanced models are able to manage many drug–drug connections and different datasets, which is necessary for use in drug safety and personalized medicine in the real world. Building such DDI prediction systems by mixing DL, graph technology, and knowledge about medicine will help pharma therapies to be made more safely and result in better healthcare outcomes.

**Figure 5:**
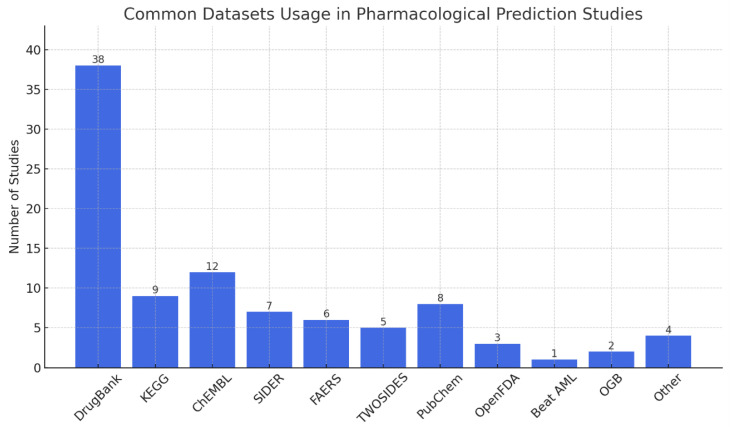
Common datasets usage in pharmacological prediction studies.

The chart in Figure 5 shows clearly that most pharmacological studies relied heavily on certain datasets. In almost half of the reviewed papers, researchers used DrugBank since it has the most comprehensive information about drugs and their effects. Other major datasets that are commonly accessed are ChEMBL, KEGG, and PubChem, supplying a lot of data about chemicals, genomes, and biology. It is common to use SIDER, FAERS, and TWOSIDES to find data on side effects and ADEs. The variety in datasets highlighted that pDDI involves different aspects, including chemicals, biological pathways, and their effects on patients.

**Figure 6:**
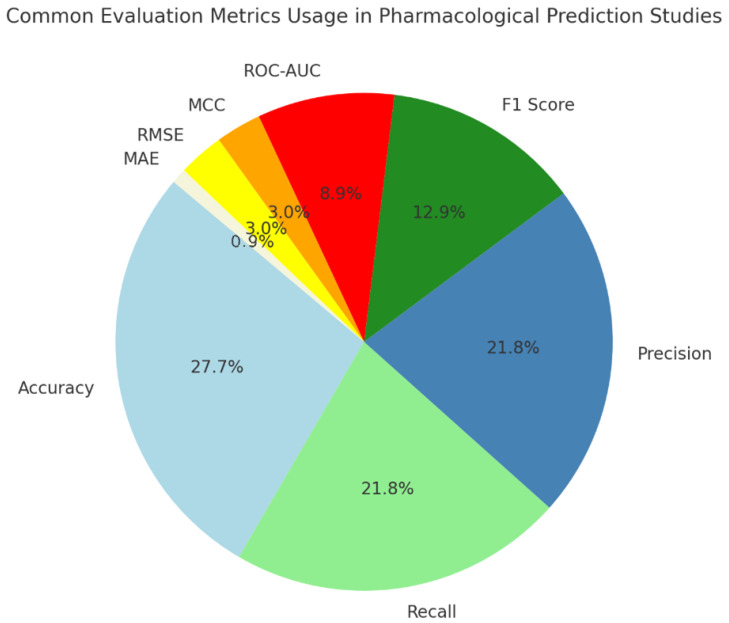
Common evaluation metrics usage in pharmacological prediction studies.

A pie chart (Figure 6) of AUC reports that accuracy is frequently used as the main evaluation tool in such studies since it is easy to understand and apply. Nevertheless, people also rely on recall, precision, and F1-score a lot in these tasks because they want to ensure both sensitivity and specificity are held in check. ROC-AUC helps us see that checking your model’s discrimination, along with its accuracy, is very useful. The metrics MCC, RMSE, and MAE are mostly used in specific areas. All things considered, using many metrics shows that assessing these models is not simple since they must work for various reasons that matter in healthcare.

PPV shows the likelihood that a model’s prediction of a positive event is correct. It is very useful in clinical situations, as the penalties of false alarms can be high. To give an example, Refs. [9,17,23] measured model accuracy by computing PPV in pDDI.

**Figure 7:**
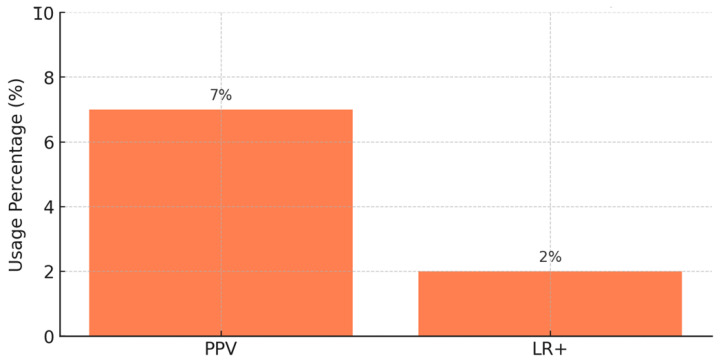
Usage of clinical metrics in pharmacological studies.

A column chart in Figure 7 made by this study illustrates how much both PPV and LR+ are used in 77 studies of pharmacological prediction. About 7% of the studies that treated this topic report the PPV.

Popular among only about 2% of studies, LR+ shows how finding a positive test result boosts the possibility of a true interaction with a drug. It is helpful for determining possible risks and making a diagnosis in clinical pharmacology. Such studies as [36,48,49] make use of LR+ to help understand how their pharmacokinetic models relate to treatment and dosing in clinical settings. Even though PPV is reported more often than LR+, both values are useful for evaluating the usefulness and reliability of pharmacological prediction models in a clinical setting. ROC-AUC is used more often instead of F1-score since F1-score is designed for situations requiring very accurate clinical support.

In Table 4, the analysis shows that there is always a trade-off between the difficulty of a DDI model, how easy it is to interpret, and its effectiveness. With the earlier ML approach, it is easy to understand, the results make sense, and overall accuracy is solid, but it does not work well for drugs with many interactions. They enhance both how accurate and how powerful a model is, but they make the model less transparent. DL and GNNs show excellent results by modeling both molecules and relationships once they are paired with transformers for the highest ROC-AUC performance (>0.90). Hybrid multimodal models help make the system more robust by linking various data sources, at the same time increasing the complexity and level of data needed. These models help provide useful information for clinical and regulatory uses, but they cannot work well with large amounts of data. Comparatively, fuzzy logic and rule-based systems explain themselves very well and handle uncertainty properly, demand manual knowledge engineering, and are not flexible.

### 4.2. A Brief Overview of Approached Data, Models, and Applications

#### 4.2.1. Data Sources

The knowledge of DDIs is based on various types of data:

- *Biological data*: Specifically, biological data refers to metabolisms, relations between gut microbiota and other microbiota, and protein–protein relations.
- *Chemical data*: These may include molecular descriptors such as SMILES, molecular fingerprints such as ECFP, and structural properties.
- *Pharmacokinetic data*: This refers to absorptive ability, metabolism, drug concentration, and pharmacodynamic data—FAERS and TWOSIDES data.

#### 4.2.2. Modeling Methods

*i.*  Traditional ML-based models are LR, SVM, and RF. The methods’ interpretability and improved efficiency have a significant impact on small-scale databases.
*ii.*  DL architectures leverage complex patterns in large-scale data:
  - *DNNs*: Incorporate nonlinearity in high-dimensional dataset.
  - *LSTM networks*: Work on sequences in any dimension (for example, concentration of drugs over time).
  - *Autoencoder*: Data compression which keeps the highlights of the dataset, i.e., compact representation.
*iii.*  Graph-based techniques are able to exploit the inherent structure of other networks of drug interactions.
  - *GCNs and GATs*: Able to encode the relationships between the drugs, targets, and diseases.
  - *Knowledge Graphs*: Represent a position of semantic relations (e.g., DrugBank and KEGG).
*iv.*  Ensemble methods combine two or more models (SVM + RF + DNNs) to improve performance and reliability. Some are gradient boosting, and the last two are stacked classifiers.

#### 4.2.3. Applications and Challenges

*i.*  Main applications:
  - *Drug safety*: Early detection of adverse interactions.
  - *Patient care*: Exploring ways to make the appropriate treatment regimens more suitable to the patient.
  - *Diagnostic decision support*: Integration with clinical workflows (e.g., EHR systems).
*ii.*  Main challenges:
  - *Data quality*: Heterogeneity and incompleteness of datasets.
  - *Scalability*: Usage of graph-based and DL models to solve complex problems increases the computational load.
  - *Interpretability*: Balancing accuracy with clinical explainability.

#### 4.2.4. Selection and Evidence Limitations

Based on the significant heterogeneity in terms of models/datasets/outcome measures, we did not conduct a meta-analysis; hence, reporting is in a narrative and tabular synthesis manner. We did not conduct a formal risk-of-bias assessment (such as ROBIS or PROBAST); rather, we used a risk of overfitting, insufficient external validation, and incomplete reporting qualitatively by weighing such risks when forming insights. Moreover, five reports were not found, which might create bias in selection, and the restriction to the English language may hamper generalizability. These limitations must be taken into consideration when making comparisons between model families and extrapolating clinical deployment.

### 4.3. Clinical Translation and Deployment: Dataset Limitations and Model Challenges

Predictive models for pDDIs, such as ML, DL, and GNNs, can enhance clinical decision-making by alerting healthcare providers to high-risk drug combinations [35,71].

XAI improves model transparency and maintains state-of-the-art performance. The integration of GCNs with attentive neural layers helps the model to highlight atoms and molecular substructures that contribute to pDDI. These visual explanations help clinicians to understand why a drug pair is predicted to interact while supporting pharmacokinetic or pharmacodynamic validation [30,44,71]. In clinical deployment, using key features that trigger alerts can help clinicians to see the likelihood of a DDI and decide if an intervention is needed. The trade-offs include false positives, which may lead to unnecessary changes in therapy, and false negatives, which could miss unsafe interaction. As a result, interpretable alerts that are trustworthy and help the clinical workflow are necessary [47,63,75,78].

Deployment of pDDI models in clinical settings requires following regulatory standards. This includes rigorous validation and model integration with pharmacovigilance systems to ensure patient safety [48,49,53,54]. While many models demonstrate strong performance on the presented datasets, such as DrugBank, TWOSIDES, and FAERS, a recent study highlights that just a few have been prospectively validated, and they have limited generalizability of pDDI in real-world applications [15]. These datasets contain inconsistencies, incomplete annotations, and contradictory DDI labels.

Additional challenge include class imbalance, where DDIs have a small portion of all possible drug pairs, forcing researchers to generate pseudo-negative samples [7,25]. These may include unstudied drug pairs with increased bias [9]. Model evaluation is complicated by data leakage [72], which occurs when a random drug-pair splitting uses chemically similar related drugs in both training and test sets. Curated databases have strong performance but lack external validation [9], and a limited clinical translation is made because DDI models mostly rely on internal splits only [15,25].

The main challenges in clinical deployment include data heterogeneity, particularly in EHRs, and the need for interpretable outputs. Future research should focus on novel approaches that combine transparent predictive performance with multi-source data integration to evaluate clinical impact [32,45,46].

## 5. Conclusions

This comprehensive review details important improvements in using ML, DL, graph-based systems, and ensemble approaches for foreseeing DDI. Because of their excellent reliability and scalability, DNNs and GNNs are considered valuable tools in clinical and pharmacological work. Even so, it is still tough for them to analyze large or low-quality sets of facts. Although ensemble and KG-based methods lead to better results, they are not easy to set up and they take increased effort in terms of computing power. It is still challenging, with aspects such as how consistent the data is, how people understand ML models, and how fast computations can be. More work should be carried out to bring in diverse knowledge, make AI models clearer, and optimize DDI prediction. Using biomimetic principles offers promising future directions to improve model training efficiency. By emulating relational adaptation, the models can represent drug interactions more accurately. As a result, the development of drugs will be safer, and drugs will be more effective and will support informed medical decisions, all of which help improve patients’ well-being and health service quality by using biomimetics to guide smarter model design.

## Figures and Tables

**Table 1:** A review of different DDI prediction methods is provided.

Study	Data	Method	Results/Aim
Comparative ML vs. DL [11]	Curated DDI pairs; bioactivity and chemical-structure features (DrugBank, ChEMBL)	LR; SVM; RF; fully connected DNN	DNNs learn nonlinear patterns and excel over ML in high-dimensional feature sets; ML is less expensive to train and suitable for low-data or low-time baselines.
Resilience to Noise [12,17]	DDI datasets perturbed with missing values and label noise; features include drug similarity, substructures, and side effects	Autoencoder-assisted DNN combined with feed-forward NN	Autoencoders regularize inputs and allow learning to stabilize in moderate noise; models perform well but require extra memory and time.
Label vs. Substructure [13,16]	FDA drug labels (PK sections, warnings, Adverse Drug Events (ADEs)); molecular fingerprints/graphs (DrugBank/ChEMBL)	Pipeline 1: label-feature extraction → NN; Pipeline 2: graph/fingerprint vectors → NN	A combination of label-derived features and chemistry-derived features outperforms separately; label features require NLP preprocessing; fingerprints are computationally expensive.
Multi-Label LSTM + ADE [18]	Time-series ADE data (e.g., concentration/exposure changes over time)	Autoencoder for dimensionality reduction, then LSTM for multi-label classification	Captures temporal patterns; enhances macro-F1 vs. static models; key temporal features preserved after ADE compression.
Ensemble Deep Models [19]	Defined DDI attributes; sequences describing how interactions unfold	Ensemble combining DNN (structure), CNN (substructure), and recurrent neural network (RNN)/LSTM (sequence)	Ensemble is more accurate with lower false positives than any individual model; shows superior generalization and calibration.
KG-Integrated DL [21]	Drug–protein–disease KG (DrugBank, KEGG)	KG embeddings combined with a DL predictor	Reduces false positives and generalizes better through relational pathways; performance improves with rich KG coverage.
Graph-Attention Networks [22]	DDI KG with multi-typed edges	GAT with edge-type awareness	Surpasses vanilla GCN baselines on DDI tasks; attention highlights clinically plausible interaction paths.
Transfer Learning [23]	Large source DDI corpora + small target dataset (scarce labels)	Pretrain on the source combined with fine-tuning on the target	Stable (AUC/F1) convergence; minimal labeled data suffices to adapt pretrained models.

**Table 2:** Overview of important DDI-related database resources.

Dataset	Type	Content	Use in DDI Modeling	Preprocessing Steps
DrugBank [60]	Pharmacological	14,000+ medicines; information on how they work, how they are handled in the body, their targets in protein molecules, and potential interactions	Improvements to add drug descriptions and model the ways they act	Removing duplicates, modifying molecular weight values, processing structures, and discarding incomplete profiles
ChEMBL [61]	Bioactivity	About 2 million compounds have been tested for bioactivity, recorded for over 15 million targets	Activity prediction and modeling interactions according to set targets	Standardizing units for biological effects, handling assay differences, removing unclear data
KEGG [62]	Pathway/Genomics	Genomic and metabolic maps for 5000 species	Studying how factors influence learning at the pathway or route level	Assigning drugs to affected pathways and adjusting graphs for modeling
FAERS [5]	ADEs	Over 10 million spontaneous reports of ADEs after marketing drugs	Comparing predictions with real-world observations	Removing duplicate entries, aligning ADEs to common definitions, linking drugs to known interactions
TWOSIDES [63]	DDI-Specific ADEs	1300 drugs, 1800+ side effects	Running DDI models using labeled adverse outcomes	Verifying each DDI pair and deleting unclear/incomplete entries
SIDER [64]	Side Effect	1400+ drugs with 5000+ side effects	Linking drug properties to possible negative effects during DDIs	Normalizing drug–effect mapping and matching with drug structure databases

**Table 3:** Comparative analysis of pharmacological task prediction models for DDI prediction. N/A—Not Applicable.

Dataset	Key References	Model Types/Methods	Datasets Used	Reported Performance	Robustness and Key Notes	Scalability & Application
Traditional ML	[6,7,12,16,17,29]	RF, SVM, DTs, LR, CatBoost, XGBoost	DrugBank, ChEMBL, KEGG, TWOSIDES, DrugComb, PubChem, FAERS, OGE	Accuracy: 85–90%, ROC-AUC: 0.85–0.90	Good baseline; struggle with nonlinear interactions; depend on feature engineering	Efficient for medium-sized datasets; easier interpretation; used in early pipelines
Ensemble Methods	[5,7,19,29,39,41]	XGBoost, CatBoost, RF, Gradient Boosted Trees	DrugCombDB, SYN-ERGXDB, Beat AML, Open Graph Benchmark, DrugBank	ROC-AUC: 0.88–0.92	Robust to noise; handle heterogeneous data; combine multiple weak learners	Scalable to large structured datasets; popular for tabular bio-data
Deep Learning	[12,13,17,18,19,43,53,54]	DNNs, CNNs, LSTMs, Transformers, Autoencoders, BiLSTM	DrugBank, ChEMBL, PubChem, SIDER, TWOSIDES, Biomedical Literature	Accuracy/ ROC-AUC: 87–92%	Capture complex interactions; need large training data; less interpretable	High scalability; computationally intensive, but flexible across data modalities
Graph-Based Models	[4,22,26,39,48,49,50,51,65,68]	GADNN, GAT, KG Embedding, GCN	DrugBank, PubChem, KEGG, SIDER, TWOSIDES, Open Graph Benchmark	ROC-AUC: 0.90–0.92	Model relationships and molecular graphs naturally; often provide interpretability	Scalable with graph optimization; best for relational and network data
Transformer Models	[24,43,48,52,53]	Transformers, DDI-Transform, RTs, PTB-DDI	DrugBank, SIDER, TWOSIDES, Biomedical Texts	ROC-AUC: 0.91–0.93	State of the art for sequence and graph data; capture contextual info well	Scalable with pretraining; highly flexible; suited for multimodal data
Hybrid and Multimodal	[26,28,30,48,49,64]	Combining Graph, Text Mining, Biomedical KG, Multimodal Fusion	DrugBank, Biomedical Text, PubChem, SIDER, FAERS	ROC-AUC: 0.90–0.92	Robust fusion of data types improves prediction and explainability	Scalable with multi-source integration; complex pipelines but powerful
Pharmaco-kinetic and PBPK Models	[55,56,58,59,60,61,62,73,77]	PBPK Models, Enzyme and Transporter Networks	Clinical trial datasets, DrugBank, FDA reports	Predict PK parameters; used for interaction mechanism evaluation	Highly interpretable, but limited to specific drugs and requires detailed parameters	Used for simulation and clinical decision support; less scalable for broad DDI
Fuzzy Logic and Rule-Based	[54,79]	Fuzzy Logic Models, Signal Detection Algorithms	DrugBank, Spontaneous Reporting Databases	Accuracy: 87–89%	Handle uncertain and complex interactions; explainable but less flexible	Scalable for rule-based knowledge systems; often combined with ML/DL methods
Data and Resources	[34,35,36,38,40,42,79]	Large Databases and Resources	DrugBank, ChEMBL, KEGG, FAERS, SIDER, nSIDES	N/A	Crucial for training, validation, and real-world deployment	Large-scale data supports advanced models; ongoing updates improve robustness

**Table 4:** Strengths and limitations of DDI prediction approaches.

Approach	Data Used	Methods Used	Performance (Accuracy/ROC-AUC)	Strengths	Limitations
Traditional ML	DrugBank, ChEMBL, KEGG, TWOSIDES, SIDER, FAERS	RF, SVM, DT, XGBoost	Accuracy: 85–90%, ROC-AUC: ∼0.85–0.90	- Easy to implement and interpret - Good baseline performance - Efficient for tabular data	- Limited in capturing complex nonlinear relationships - Heavily dependent on feature engineering
EM	DrugComb, Beat AML, DrugBank, Open Graph Benchmark	XGBoost, CatBoost, Gradient Boosting Trees	ROC-AUC: 0.88–0.92	- Robust to noise and overfitting - High predictive accuracy - Handles heterogeneous data	- Interpretability can be reduced - Less effective on graph or sequence data
DNNs	DrugBank, ChEMBL, SIDER, PubChem, TWOSIDES	CNN, LSTM, Transformer, Autoencoder, BiLSTM	Accuracy/ROC-AUC: 87–92%	- Captures complex drug interactions - Learns features automatically - Flexible architectures	- Requires large datasets - Computationally expensive - Often less interpretable
GNNs	DrugBank, KEGG, PubChem, SIDER, TWOSIDES	GCN, GAT, KG Embedding	ROC-AUC: 0.90–0.92	- Models drug interactions as graphs naturally - High interpretability - Handles multimodal data	- Scalability can be challenging - Requires well-curated graph data
Transformer Models	DrugBank, Biomedical Texts, SIDER, TWOSIDES	DDI-Transformer, RTs	ROC-AUC: 0.91–0.93	- Excels in sequence and context modeling - State-of-the-art accuracy - Good TL	- High computational cost - Complex training and tuning
Hybrid and Multimodal Models	DrugBank, the Biomedical Literature, PubChem, FAERS	Fusion of Graph, Text Mining, and DL	ROC-AUC: 0.90–0.92	- Integrates diverse data sources - More robust and generalizable - Improves interpretability	- Complex architectures - Data integration challenges - Requires large, diverse datasets
PBPK Models	Clinical Trial Data, FDA reports, DrugBank	Mechanistic PK Modeling, Enzyme/Transporter Networks	Performance: mechanistic evaluation, simulation accuracy	- Provides clinical interpretability - Predicts drug metabolism and DDI mechanisms - Valuable for regulatory decisions	- Requires detailed drug parameters - Limited scalability - Not suited for large-scale screening
Fuzzy Logic and Rule-Based Models	DrugBank, Spontaneous Reporting Databases	Fuzzy Logic, Rule-Based Systems, Signal Detection	Accuracy: ∼87–89%	- Handles uncertainty and incomplete data well - High explainability - Useful in clinical settings	- Less flexible - Rule creation labor-intensive - May miss unknown interaction patterns

## Data Availability

No data was used in this study.

## Abbreviations

The following abbreviations are used in this manuscript:

ADE	Adverse Drug Events
ADME	Absorption, Distribution, Metabolism, and Excretion
ADRs	Adverse Drug Reactions
AI	Artificial Intelligence
ChEMBL	Chemical Database of Bioactive Molecules
DAS-DDI	Dual-View Framework with Drug Association and Drug Structure for pDDI
DTs	Decision Trees
DL	Deep Learning
DNN	Deep Neural Network
DDIs	Drug–Drug Interactions
DSIL-DDI	Domain-Invariant Substructure Interaction Learning for pDDI
EHR	Electronic Health Record
EM	Ensemble Methods
FAERS	FDA Adverse Event Reporting System
FDA	Food and Drug Administration
GADNN	Graph Attention-based Deep Neural Network
GATs	Graph Attention Networks
GBTs	Gradient Boosted Trees
GCNs	Graph Convolutional Networks
GNNs	Graph Neural Networks
HTCL-DDI	Hierarchical Triple-View Contrastive Learning Framework for pDDI
KEGG	Kyoto Encyclopedia of Genes and Genomes
KNN	K-Nearest Neighbors
LR	Logistic Regression
LR+	Positive Likelihood Ratio
LSTM	Long Short Term Memory
MCC	Matthews Correlation Coefficient
ML	Machine Learning
NLP	Natural Language Processing
nSIDES	Drug Side Effects and Interactions
PBPK	Physiologically Based Pharmacokinetic
pDDI	Prediction of Drug–Drug Interaction
PEB-DDI	A Task-Specific Dual-View Substructural Learning Framework for pDDI
PPV	Positive Predictive Value
PR-AUC	Precision–Recall–AUC
PTB-DDI	Pretrained Tokenizer and BiLSTM Model for pDDI
PU	Positive Unlabeled
RF	Random Forest
ROC-AUC	Receiver Operating Characteristics Curve–Area Under the Curve
RNNs	Recurrent Neural Networks
RTs	Relational Transformers
RQs	Research Questions
SIDER	Side Effect Resource
SMILES	Simplified Molecular Input Line Entry System
SSF-DDI	Drug Sequence and Substructure Features for pDDI
SubGE-DDI	Subgraph Enhance Model for pDDI
SVMs	Support Vector Machines
TL	Transfer Learning
TWOSIDES	Towards Understanding Side Effects of Drugs for Healthcare Data Analytics
XAI	Explainable AI
